# Multilevel Reset Dependent Set of a Biodegradable Memristor with Physically Transient

**DOI:** 10.1002/advs.202306206

**Published:** 2023-11-30

**Authors:** Mohammad Tauquir Alam Shamim Shaikh, Tan Hoang Vu Nguyen, Ho Jung Jeon, Chowdam Venkata Prasad, Kyong Jae Kim, Eun Seo Jo, Sangmo Kim, You Seung Rim

**Affiliations:** ^1^ Department of Semiconductor Systems Engineering and Institute of Semiconductor and System IC Sejong University Seoul 05006 Republic of Korea; ^2^ Department of Intelligent Mechatronics Engineering and Convergence Engineering for Intelligent Drone Sejong University Seoul 05006 Republic of Korea

**Keywords:** biocompatible, multilevel memory, polyvinylpyrrolidone, resistive switching, transient memory

## Abstract

The electronic device, with its biocompatibility, biodegradability, and ease of fabrication process, shows great potential to embed into health monitoring and hardware data security systems. Herein, polyvinylpyrrolidone (PVP) biopolymer is presented as an active layer, electrochemically active magnesium (Mg) as a metal electrode, and chitosan‐based substrate (CHS) to fabricate biocompatible and biodegradable physically transient neuromorphic device (W/Mg/PVP/Mg/CHS). The *I*–*V* curve of device is non‐volatile bipolar in nature and shows a unique compliance‐induced multilevel RESET‐dependent‐SET behavior while sweeping the compliance current from a few microamperes to milliamperes. Non‐volatile and stable switching properties are demonstrated with a long endurance test (100 sweeps) and retention time of over 10^4^ s. The physically transient memristor (PTM) has remarkably high dynamic R_ON_/R_OFF_ (ON/OFF state resistance) ratio (10^6^ Ω), and when placed in deionized (DI) water, the device is observed to completely dissolve within 10 min. The pulse transient measurements demonstrate the neuromorphic computation capabilities of the device in the form of excitatory post synaptic current (EPSC), potentiation, depression, and learning behavior, which resemble the biological function of neurons. The results demonstrate the potential of W/Mg/PVP/Mg/CHS device for use in future healthcare and physically transient electronics.

## Graphical abstract

Polyvinylpyrrolidone (PVP) biopolymer as an active layer, electrochemically active magnesium (Mg) as a metal electrode, and chitosan‐based substrate (CHS) to fabricate biocompatible and biodegradable physically transient neuromorphic device (W/Mg/PVP/Mg/CHS).

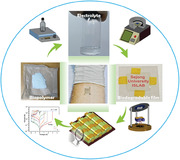

## 1 Introduction

Increasing the memory density of the current complementary‐metal‐oxide‐semiconductor (CMOS) technology is the key challenge faced by researchers due to the physical and fundamental limitations of Von Neumann architecture.^[^ ^1^ ^]^ One solution to this limitation is to stack the devices on a 3D plane rather than a 2D plane. However, this stacking process poses several challenges, including high fabrication cost and complexity, fabrication yield, sneak path issues, and reliability concerns.^[^ ^2^, ^3^ ^]^ In order to expand multi‐bit storage capacity without sacrificing additional 2D area, it has been shown that resistive switching random access memory (RRAM) is capable of demonstrating multilevel memory states by modulating its internal state parameters.^[^ ^4^, ^5^ ^]^ The RRAM is one of the classes of memory concepts that is based on the electrical stimulation‐induced change in resistance of a metal‐insulator‐metal (MIM) memory structure.^[^ ^6^, ^7^ ^]^ The crude classification of RRAM devices is based on the working principle, in which one device operates entirely on the migration and redistribution of cations, such as Cu or Ag, Mg, and Al, etc. ions, while another is based on the migration of anions, where oxygen ions or oxygen vacancies play a major role.^[^ ^8^, ^9^, ^10^ ^]^ RRAM devices, when subjected to physically transient electronic fields, can exhibit complete biocompatibility with human tissue and have the ability to dissolve when no longer needed. These characteristics make RRAM promising candidates for future applications such as implantable bioelectronics, biomedical diagnostics, and secure hardware systems.^[^ ^11^, ^12^ ^]^

Researchers around the world are utilizing organic materials to reduce the generation of electronic waste and avoid the high cost associated with cycling or disposing of discarded electronics.^[^ ^13^ ^]^ Glucose bipolar resistive switching characteristics and biocompatibility have been demonstrated by Kim et. al.^[^ ^14^ ^]^ The glucose device shows a good switching window of 10^3^ and maintains stability for 100 consecutive cycles. The design and use of protein nanocages to tune the conductance inside the nanogap device have achieved different states.^[^ ^15^ ^]^ The multilevel switching and fast switching speed were revealed in a pectin‐based biomaterial derived from orange peel waste.^[^ ^16^ ^]^ The gradual and abrupt change in the current level was observed in the starch and starch‐chitosan mixed biopolymer during controlling the resistive switching behavior.^[^ ^17^ ^]^ While synthetic ion‐conducting polymers have also exhibited remarkable performance in biocompatible and biodegradable devices, as demonstrated in letters.^[^ ^18^, ^19^ ^]^ The gold‐decorated chitosan‐based facile solution processed flexible device demonstrated resistive switching behavior with biocompatibility.^[^ ^20^ ^]^ The fabricated device showed programmable non‐volatile memory characteristics under tensile and compressed bent states.

In our study, we used a synthetic polymer called polyvinylpyrrolidone (PVP), which consists of 1‐vinyl‐2‐pyrrolidone monomers and has both π and σ bonds of different lengths,^[^ ^21^ ^]^ to create a solid polymer electrolyte with good ionic transport properties.^[^ ^9^ ^]^ Also, the polymers are of ease of thin film forming, good adhesiveness on various substrates, good mechanical strength, and low fabrication costs.^[^ ^10^ ^]^ One of the most important properties of the active layer in resistive switching devices is its high ionic conductivity, which allows for the conduction of metal ions in filamentary‐type RRAM.^[^ ^7^, ^22^ ^]^ Apart from its high ionic conductivity,^[^ ^23^ ^]^ PVP can dissolve in water as well as other polar solvents^[^ ^21^ ^]^ and has good interactive ability with metal ions to be used as an active layer in ReRAM devices.^[^ ^24^ ^]^ The Xu et.al. used PVP and N‐doped carbon quantum dot nanocomposite in a resistive switching device for realizing neuromorphic computing behavior.^[^ ^25^ ^]^ The device exhibited charge trapping/detrapping functionality as the conduction mechanism, as well as good short‐term/long‐term plasticity (STP/LTP) that was emulated by applying a pulse signal. Dlamini et al. compared the resistive switching behavior in composite‐based memory devices by using different active layers and top electrode configurations.^[^ ^26^ ^]^ Yang et al. choice of ion transfer material was an important factor in the determination of the nucleation side.^[^ ^23^, ^27^ ^]^ The studies have revealed the potential of polyvinylpyrrolidone as an active layer in resistive switching devices for fundamental research.^[^ ^28^ ^]^ However, being a biocompatible and biodegradable polymer (PVP), the existing work is not inclined toward high‐density memory devices with transient properties. Also, as in the active layer, PVP has not shown multilevel states and high ON/OFF ratio even though it has a hybrid structure of organic/inorganic materials.^[^ ^29^, ^30^ ^]^ Considering the biodegradable properties of magnesium metal and its favorable electrochemical characteristics, it has shown the potential to reduce the SET/RESET voltage in RRAM.^[^ ^31^ ^]^ Herein, we effectively utilized the chemical, electrochemical, and biocompatibility properties of PVP and magnesium metal to enable multilevel RESET‐SET operations in the device. Furthermore, integrating chitosan substrate with the RRAM device demonstrates its potential for use in future biocompatible wearable electronics. Therefore, the physically transient memristor (PTM) not only exhibited excellent biocompatibility but also demonstrated promising potential for use in high‐density memory devices.

## 2 Results and Discussion

### 2.1 PTM Resistive Switching Characteristics

**Figure 1:**
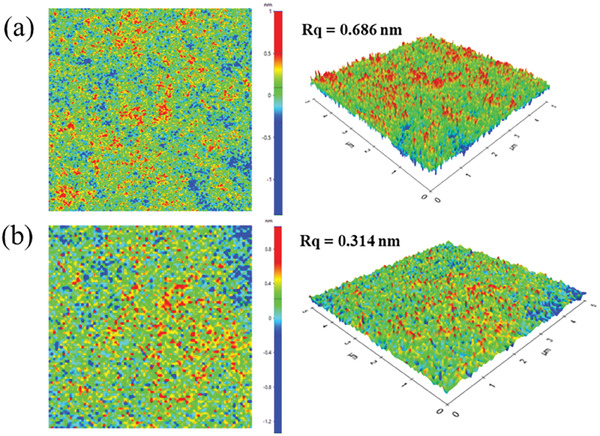
Shows an atomic force microscopy (AFM) image of the topology and roughness of the PVP active layer. a) Dried at room temperature and atmospheric pressure. b) Dried in vacuum (10^−3^ mbar), having a root‐mean‐square (RMS) roughness value of 0.686 and 0.314 nm respectively, scanning area of 5 × 5 µm^2^.

**Figure 2:**
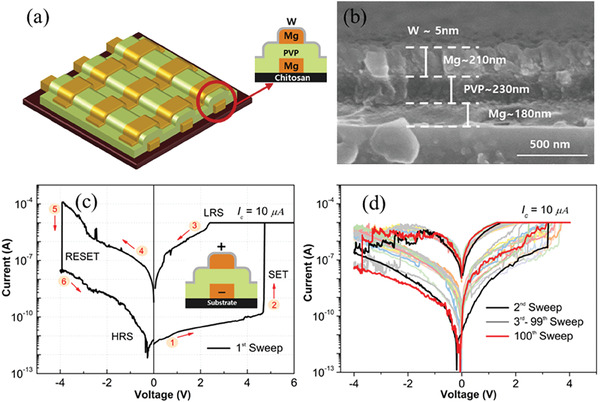
Reveals the W**/**Mg/PVP/Mg/CHS memristor. a) 3D structure schematics and inset image showing cross‐section view of same. b) Cross‐sectional scanning electron microscope (SEM) image depicting different layer thicknesses. c) Forming *I*–*V* sweep and schematic of cross‐section view for biasing the device. d) The endurance characteristics of the device were evaluated by running 99 consecutive *I*–*V* sweeps.

The active layer of the resistive switching device plays a crucial role in both filament formation and controlling the resistive switching characteristics.^[^ ^7^, ^32^ ^]^ As the low roughness and amorphous nature of the active layer help reduce the average and standard deviation values of set and reset voltages through good ionic transportation and confining the filament to the smallest active area,^[^ ^14^ ^]^ we optimized the roughness of the active layer using the vacuum drying process.^[^ ^33^ ^]^ After the spin coating process, the film was placed in the vacuum oven overnight, resulting in a roughness change from 0.686  to 0.314 nm for non‐vacuum and vacuum‐dried film, as shown in AFM images **Figure** **1**a,b, respectively. The low roughness may come from shrinking the free volume of the polymer film during the vacuum drying process.^[^ ^34^ ^]^ The XRD spectrum of the prepared PVP film showed a broad peak present at 12–18 °C degrees, which indicates the inherent amorphous nature^[^ ^35^ ^]^ of the active layer (Figure S1a, Supporting Information). The FESEM morphology analysis also shows homogenous film formation over the entire surface area (Figure S1a, Supporting Information). A 3D schematic of the device configuration, including the active layer and its edge formation due to the bottom electrode (**Figure** **2**a). The complete fabrication process of PTM (W/Mg/PVP/Mg/CHS) has been described in detail in the Experimental Section, and a schematic representation of the same has been provided in Figure S2a–e (Supporting Information). The device was fabricated on chitosan‐based biocompatible and biodegradable substrate (Figure S3a,b, Supporting Information) in order to have complete physically transient properties. In the MIM structure depicted in the inset image of Figure 2a, the magnesium (Mg) in the top and bottom electrodes serve as electrochemically active electrodes, while the PVP film acts as an active layer. Furthermore, to protect the top magnesium electrode from chemical and mechanical damage in corrosive or abrasive environments, an inert tungsten (W) thin layer was deposited on top of it as a protective media. Figure 2b shows a cross‐sectional image obtained by FESEM revealing all the distinct layers of the device: the bottom electrode (≈180 nm), active layer (≈230 nm), and top electrode (≈210 nm), while (Figure S3a,b, Supporting Information) displays a photograph of the biodegradable substrate and complete crossbar array device.

Figure 2c represents the semi‐logarithmic plot of the first sweep applied to PTM in 10 × 10 arrays. The device has non‐volatile behavior, which comes when a positive voltage is applied to the top magnesium (Mg) electrode with respect to the bottom electrode (inset Figure 2c). The device switched from its initial OFF state also known as the high resistance state (HRS) to the low resistance state (LRS) at 4.71 V and stayed in the LRS even though the voltage was brought to 0 V. Subsrquently the device returned to its initial OFF state (HRS) at −3.93 V on applying negative voltage. This completes the full set and reset operation of the device, setting the benchmark for further investigations. The 10 µA compliance current was applied to restrict the excessive filament growth and to form a quantized conductance contact (n = G/G0, where n = 1, 2, 3… and n = 1 represents the atomic point contact, G = I/V, G0 = 2e^2^/h = 12.9 kΩ, e is the electron charge and h is plank's constant) across the active layer. The effect of this compliance is explained in the upcoming section. To check the stability of the set‐reset operation on the same cell, the device was swept 100 times continuously with positive and negative voltage (Figure 2d). The device demonstrates stable butterfly *I*–*V* characteristics in the voltage range of 0 to 4 V and 0 to ‐4 V during the SET and RESET, respectively. After the first forming process and subsequent sweeps, the SET voltage decreases as the active magnesium metal ions dissolve and diffuse into the polymer matrix.^[^ ^36^ ^]^ Similarly, after the first RESET, the subsequent RESET was reduced to below the first RESET voltage. This may be due to the partial breakage of the multifilament during the first forming reset sweep and the presence of residual unruptured filaments inside the amorphous active layer.^[^ ^27^, ^37^ ^]^ These long “SET‐RESET‐SET‐RESET” operations with high switching stability show an increase in resistance at the end of the endurance sweeps, which in turn increases the SET voltage during the intermediate sweeps.

**Figure 3:**
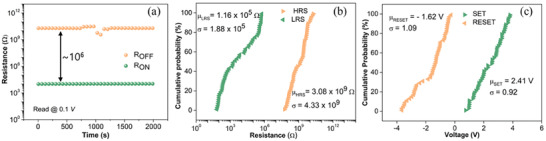
a) ON (LRS) and OFF (HRS) retention characteristics at *I*_CC_ = 10^−5^ A and a memory window of 10^6^. Cumulative Probability distribution for 100 sweeps of. b) resistance of LRS and HRS. c) SET and RESET voltage.

The memory window (R_ON_/R_OFF_ ratio) of the HRS and LRS is large ∼10^6^ and shows no degradation in retention states for up to 2 × 10^3^ s (**Figure** **3**a).^[^ ^38^ ^]^ A statistical distribution of both resistance states (R_LRS_/R_HRS_) and switching voltages (V_SET_/V_RESET_) was directly extracted from the endurance sweeps (Figure 2d). It was observed that all the mean (µ) values of R_LRS_ (1.16 × 10^5^ Ω) and R_HRS_ (3.08 × 10^9^ Ω) scatter to a certain extent with a standard deviation (σ) of 1.188 × 10^5^ and 4.33 × 10^9^ respectively (Figure 3b). Similarly, V_SET_/V_RESET_ scattering was also recorded in terms of mean (µ) values of 2.41 V and −1.62 V with a standard deviation (σ) of 0.92 and 1.04, respectively (Figure 3c). The scattering of HRS and LRS is common in very large window memory (ON/OFF ratio), and it helps to tune the device into multilevel states.^[^ ^39^, ^40^ ^]^

### 2.2 Conduction Mechanism in PTM

To understand the mechanism of current conduction and kinetics of filament formation in the device log (I)‐ log (V) was plotted (Figure S4a, Supporting Information). The curve exhibits three main fitting regions with slope values varying from 1 to 2 and >2, suggests two distinct basic conduction mechanisms.^[^ ^7^, ^41^, ^42^ ^]^

Space‐charge‐limited‐current (SCLC) conduction model:

(1) $$I\;\propto \;V^{\alpha }$$

Ohmic conduction model:

(2) $$I\;\propto V\;\exp\left(-\Delta E_{e}/kT\right)$$

where I, V, α, E_e_, k, and T are current, voltage, slope constant, activation energy of electrons, Boltzmann's constant, and absolute temperature, respectively.

The linear fitting during the SET process has a slope value (α) of 1.5 (violet line), which converges to 2 (red) and relates to the SCLC mechanism.^[^ ^7^ ^]^ The space charge is majorly generated due to defects and the early reduction of Mg^2+^ inside the active layer,^[^ ^43^ ^]^ before they are reduced at the bottom electrode.^[^ ^44^ ^]^ However, if the bias voltage is increased further, the electron energy increases to a level where it can overcome all the barriers, tunnel through the polymer matrix, and get trapped in the defect sites of the polymer matrix.^[^ ^45^ ^]^ As the trap sites are filled and the voltage is further increased to the higher side, the device current abruptly increases, turning the device from HRS to LRS state (Figure S4a, Supporting Information). The slope value before it goes into LRS is >2, which shifts the conduction mechanism from SCLC to trapped charge‐limited current (TCLC) (for all α >2). The conduction in the LRS is dominated by ohmic behavior, as shown with α = 1 (green).^[^ ^46^ ^]^ The slope (α) during reset sweep has values of 2.7, 2, and 1 (blue, red, and orange lines, respectively), indicating a transition from SCLC to an ohmic conduction mechanism.^[^ ^47^ ^]^ The multilevel reset (Figure S4b, Supporting Information), follows similar slope values for HRS1, HRS2, HRS3, HRS4, and HRS5, indicating a consistent transition from HRS to LRS after each reset sweep. All the states have slope values from 1 to ∼1.8 and >2 indicating SCLC and trap filled regions.

**Figure 4:**
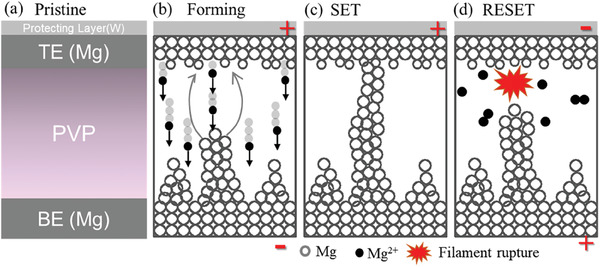
Represents a schematic of the electrochemical metallization (ECM) process in W/Mg/PVP/Mg/CHS memristor. a) The pristine state of the PTM. b) Oxidation, migration, and reduction of Mg^2+^ ions in the polymer matrix during the SET process. c) Reduction and accumulation of Mg atoms on the bottom electrode, which leads to the growth of highly conducting filament. d) Filament rupturing and Mg atom dissolution inside the polymer matrix during the RESET process.

Based on the linear fitting data (Figure S4a, Supporting Information) and reported metal/biopolymer RRAM devices,^[^ ^18^, ^44^ ^]^ a simple schematic has been drawn to further elaborate the electrochemical metallization (ECM) process (**Figure** **4**a). Applying a positive voltage to the Mg top electrode leads to the electrochemical oxidation of magnesium metal (Mg → Mg^2+^ + 2e) which migrates toward the bottom electrode through the active layer (Figure 4b). The oxidized ions get reduced at bottom Mg electrode (Mg^2+^ + 2e → Mg) and hence complete the electrochemical oxidation‐reduction process. Due to continued sweep voltage, the more Mg^2+^ gets reduced on the bottom electrode, and a stack of Mg atoms reaches the top electrode (Figure 4c). At this point the device goes to LRS by increasing the current abruptly, which is known as SET process. The rupturing of filaments occurs near the interface when applying negative voltage at the top Mg with respect to the bottom electrode (Figure 4d). After applying a negative voltage, the dissociation of filaments is caused by three factors.^[^ ^48^, ^49^ ^]^ First, the oxidation reaction takes place on the formed filament. Second is the joule heating effect, which is one of the most important effects leading to thermal reset in most resistive switching. And third, the removal of the metal ions (Mg^2+^) in the formed filament due to the electric field.^[^ ^6^ ^]^

Furthermore, X‐ray photoelectron spectroscopy (XPS) depth analysis was done to check the valency of Mg metal inside the polymer layer for metallic filament validation. The intensity of the Mg 2p spectrum is high on the surface due to the metallic electrode, whereas the intensity reduces gradually as it enters the polymer layer (Figure S4e, Supporting Information). The Mg 2p spectrum could be deconvoluted to the Mg‐Mg peak (Mg^0^ oxidation state) at ≈49.6 eV which can be tracked down from the top electrode to inside the polymer layer (Figure S4f, Supporting Information).^[^ ^50^ ^]^ The Mg‐O peak centered at ≈50.7 eV was also deconvoluted and could be due to the surface oxidation of the Mg electrode on and in proximity to the PVP film, as its intensity reduces in depth compared to the Mg‐Mg peak intensity. This shows that the Mg‐Mg intensity is dominated from the top electrode surface to inside the polymer. The deconvoluted spectrum of O1s also has two peaks at ≈ 530.8 eV and ≈ 532.9 eV which correspond to Mg═O bonds and O (‐C_6_H_5_N‐H)n, respectively as reported.^[^ ^51^, ^52^ ^]^ The intensity of O (‐C_6_H_5_N‐H)n increases as it goes in polymer with high concentration (C_6_H_9_NO)n whereas the Mg═O intensity decreases considering very few Mg reacts with polymer film. From the XPS spectrum, we further confirmed that Mg is responsible for the resistive switching characteristics in our device.

### 2.3 Multilevel RESET Dependent SET Behavior

**Figure 5:**
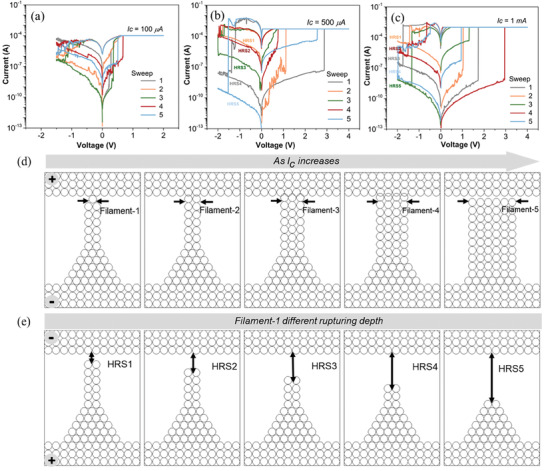
Represents the complianceinduced multilevel RESET‐SET operation of the W/Mg/PVP/Mg/CHS memristor obtained by controlling the compliance current. a) *I*–*V* curve of *Ic =* 100 µA. b) *I*–*V* curve of *Ic* = 500 µA. c) *I*–*V* curve of Ic *=* 1 mA. Schematic representation of multilevel mechanism: d) Possible filament formation and its textures during the LRS. e) Possible filament rupturing point and depth of filament‐1 for multilevel HRS during the reset.

The W/Mg/PVP/Mg/CHS memristor exhibited a butterfly *I*–*V* curve during the SET and RESET processes when the compliance current was 10 µA. The device was tuned into a multilevel HRS (HRS1, HRS2, HRS3, HRS4, and HRS5) during the RESET process by increasing the compliance current from the initial 10 to 100 µA (**Figure** **5**a). The distinct RESET states were not observed until the current was increased to 500 µA (Figure 5b). Each SET current begins exactly with the preceding RESET current left off at a voltage that is close to zero, as depicted in the 1st–5th sweep of I‐V curves. Figure 5c reveals that increasing the compliance current from 500 µA to 1 mA resulted in a more distinguishable RESET‐dependent SET behavior. This enables us to investigate the potential mechanism underlying RESET dependency in more detail. The metallic filament type resistive switching devices on multilevel RRAM^[^ ^5^ ^]^ indicate that this RESET‐dependent SET may be attributed to various factors, such as rupturing of the filament only for a few atomic layers, multiple layers, thickening of the filaments due to an increase in the compliance current, and stochastic behavior of filaments inside the active layer.^[^ ^53^ ^]^ Figure 5d demonstrates that as the compliance current increases, the filament with different diameters (Filament‐1, Filament‐2, and so on) forms which results in different ON state resistance due to changes in its area. Each of the different filaments (Filament‐1, Filament‐2, etc) can rupture at different depths, as shown for Filament‐1, which is the main cause of the multilevel RESET phenomenon in our device (Figure 5e). The breaking depth is random and difficult to control due to the stochastic nature of the filaments inside the active layer.^[^ ^53^, ^54^ ^]^

**Figure 6:**
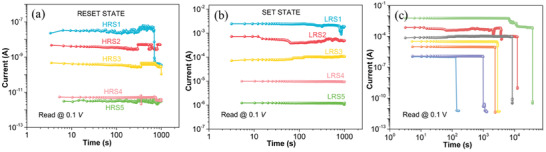
Shows the retention characteristics of PTM after each HRS and LRS. a) Real‐time retention of HRS1, HRS2, HRS3, HRS4, and HRS5. b) Real‐time retention of LRS1, LRS2, LRS3, LRS4, and LRS5. c) Real‐time degradation of multilevel memory from set to reset state.

**Figure** **6**a shows the real‐time retention of multilevel resistance states (HRS1, HRS2, HRS3, HRS4, and HRS5) for a duration of up to 1 × 10^3^s. The resistance decrease after a certain time can be observed in HRS1 and HRS2, which indicates the possibility of further rupturing of the filament. The comparative resistance values of the different states, “HRS5 > HRS4 > HRS3 > HRS2 > HRS1,” indicate that HRS5 has the highest resistance, followed by HRS4, HRS3, HRS2, and HRS1, respectively. Therefore, HRS5 has experienced the maximum depth of filament rupturing, while HRS1 has the lowest depth of rupturing, resulting in a high resistance value for HRS5 as compared to HRS1. Similarly, the LRS (Figure 6b) have distinct multilevel LRS (LRS1, LRS2, LRS3, LRS4, and LRS5) up to 1 × 10^3^ s. Furthermore, the real‐time degradation of different LRS were created by limiting the ON state current from a few µA to 1 mA (Figure 6c). As the LRS current increases, the retention time also increases. The retention time for LRS with low current compliance is very short and could be considered for short‐term memory, whereas higher current compliance could be used for long‐term memory applications.^[^ ^55^ ^]^

### 2.4 Neuromorphic Behavior of PTM

**Figure 7:**
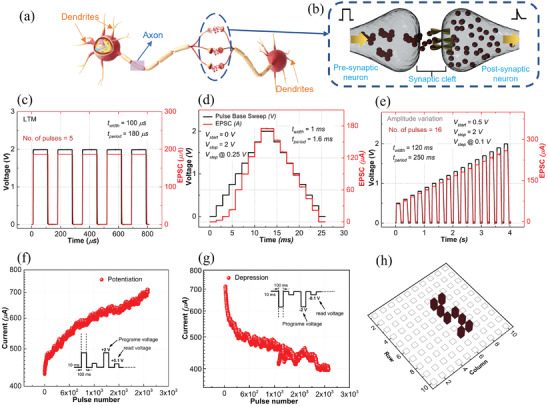
a, b) Schematic representation of the biological synapse, illustrating the pre‐synaptic and post‐synaptic neurons. Neuromorphic behavior of the PTM device: c) EPSC by repetitive pulses of the same magnitude with constant time (V_pulse_ = 2 V, t_width_ = 100 µs, t_period_ = 180 µs). d) Variation of EPSC with dual sweep pulse mode, varying the magnitude (V_start_ = 0 V, V_stop_ = 2 V_step_ = 0.25 V, t_width_ = 1 ms, t_period_ = 1.6 ms). e) EPSC modulation by changing the amplitude of pulses (V_start_ = 0 V, V_stop_ = 2 V_step_ = 0.1 V, t_width_ = 120 ms, t_period_ = 250 ms). f, g) Potentiation and depression performance of the PTM device in response to a positive and negative stream of 2 V, 100 ms pulses applied. h) The schematic of the letter “S” consists of 8 pixels generated by a training pulse to show the learning process in the brain.

To achieve the development of a neuromorphic system that has the capacity for biological‐like functions, it is imperative to produce a synapse device that fulfills prerequisites such as low energy consumption, multilevel memory functions for high density, scalability, and the capability to undergo a physical transient mode when needed.^[^ ^2^ ^]^ In our PTM device, we explored the potential of bioinspired functions, such as excitatory post‐synaptic current (EPSC) and learning behavior, in multilevel neuromorphic computing applications. **Figure** **7**a,b represent the schematic of a biological neuron and a synapse based on the W/Mg/PVP/Mg/CHS memristor, respectively. The top electrode acts as the pre‐synaptic neuron, whereas the bottom electrode acts as the post‐synaptic neuron (Figure 7b). The pulse response (Figure 7c) reveals that magnesium ions migrate through the synapse cleft to the post‐synaptic neuron on applying the consecutive electrical pulses (V_pulse_ = 2 V, t_width_ = 100 µs, t_period_ = 180 µs, and pulses = 5). The synapse current (I_EPSC_ = 185 µA) did not show much variation for all the five applied pulses, and this constant current arises due to the non‐volatile nature of the filaments.^[^ ^56^ ^]^As it can be seen in Figure 7d, we performed a dual sweep pulse‐based *I*–*V* measurement to learn more about how input voltage pulse stimuli affect EPSC. A notable observation is that the post‐synaptic current showed clear increments with the variation of the input voltage pulse. The incremental steps of the pulse (V_start_ = 0 V, V_stop_ = 2 V, V_step_ = 0.25 V, t_width_ = 1 ms, and t_period_ = 1.6 ms) provide an increasing amount of charge carriers to the synapse cleft. As a result, there is a corresponding increase in the post‐synaptic current. After applying the incremental pulses, the maximum post‐synaptic current achieved was 175 µA at V_step_ = 2 V, which is almost identical to the value shown in Figure 7c. On the other hand, the behavior of the reverse sweep pulse is characterized by a gradual drop in current, in contrast to the forward sweep pulse.

These findings suggest that the synaptic weights can be modified continuously in response to changes in base potential, which sheds light on the short‐term memory (STM) and long‐term memory (LTM) behavior of the device.^[^ ^57^ ^]^ Furthermore, the EPSC could also be modulated by pulse amplitude variation, that is, spike‐amplitude‐dependent plasticity, in which a higher positive pulse amplitude leads to enhanced EPSC and shortens the time to reach saturated post‐synaptic current. To demonstrate the LTM operation, the applied pulse width was varied (t_width_ = 120 ms, and t_period_ = 250 ms) by varying the amplitude of the pulse (V_start_ = 0 V, V_stop_ = 2 V, and V_step_ = 0.1 V) (Figure 7e). As it can be seen, the current was in line with the pulse step variation, and a gradual increase in current was observed up to V_step_ = 2 V. The increase in the final current (I_EPSC_ = 260 µA) is mainly contributed to by the increase in the pulse width (t_width_) from 1 to 120 ms, which also reveals the effect of pulse width variation on PTM. In addition to exhibiting gradual resistance switching behavior during a DC sweep, the device capable of modulating current can be programmed using a stream of consecutive pulses.^[^ ^58^ ^]^ The graph (Figure 7f,g) demonstrates that the current can be gradually modulated to simulate both long‐term potentiation (LTP) and long‐term depression (LTD) with 2000 consecutive programming pulses, indicating the potential of this synaptic device for learning and forgetting behavior. By applying consecutive positive pulses (2 V, 100 ms) followed by negative pulses (−2 V, 100 ms), the current of the synaptic device gradually potentiated and depressed. During the depression pulses, the decrease in current is initially non‐linear and is attributed to the high conductivity of the device.^[^ ^59^ ^]^ However, after a few pulses, the current starts decreasing linearly (Figure 7g). To emulate the learning behavior of PTM, a training pulse (V_step_ = 2 V, t_width_ = 100 ms) was applied (Figure S5a, Supporting Information) to the 10×10 array (Figure S5b, Supporting Information). The “S” and “I” consisting of 8 and 5 pixels, respectively, were generated and are shown in the schematic Figure 7h and Figure S5c (Supporting Information). After applying the training pulses, the synaptic current could be memorized by the individual cells in the device, which indicates the learning process in the biological brain. The presence of multiple accessible current states that increase incrementally, along with their linearity and symmetry, indicates the potential for utilizing this device in neuromorphic computing and deep neural network applications.^[^ ^3^ ^]^

### 2.5 Physically Transient Behavior of W/Mg/PVP/Mg/CHS

**Figure 8:**
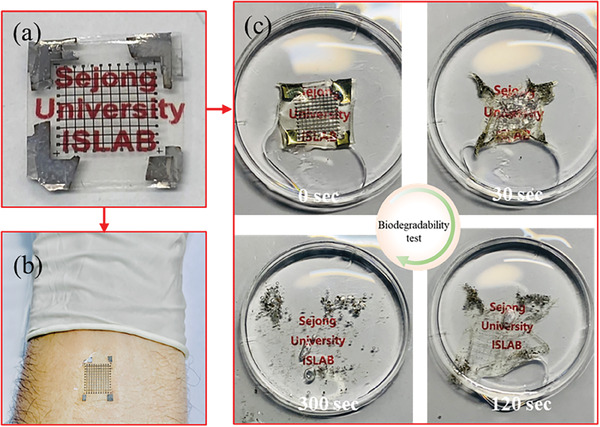
Shows photograph of a) W/Mg/PVP/Mg/CHS memristors on biocompatible and biodegradable substrate (Chitosan). b) Bio and wearable compatibility of W/Mg/PVP/Mg/CHS memristors. c) PTM biodegradability in deionized water at room temperature over various time intervals: 0, 30, 120, and 300 s.

Physically transient devices are electronic devices intentionally designed to dissolve or break down over time through physical or chemical mechanisms such as exposure to light, heat, or water.^[^ ^11^, ^12^ ^]^ The W/Mg/PVP/Mg/CHS device (**Figure** **8**a) is a flexible, transparent, and strong candidate for biocompatible wearable electronics, as depicted in Figure 8b. The PTM device's biodegradability was tested by immersing the device in deionized water at room temperature and tracking the dissolution process over several time intervals (Figure 8c). The biodegradation process of magnesium primarily involves a combination of chemical reactions and biological processes [Mg(s) + 2H_2_O(l) → Mg(OH)_2_(aq) + H_2_(g)].^[^ ^60^ ^]^ The reaction leads to the formation of magnesium hydroxide on the surface of the metal electrode and generates magnesium ions (Mg^2+^) in the surrounding water. These ions are soluble and can easily be taken up by living organisms or become part of the natural ecosystem. The top electrode (Mg) dissolves within 30 s, while the active layer, which is also a biopolymer, degrades over time (Figure 8c). The active layer (PVP) we used is a water‐soluble synthetic polymer (C_6_H_9_NO)n having low molecular weight (10 000) for easy and quick degradation. When PVP is exposed to water, the water molecules interact with the polymer chains, leading to the hydrolysis of the ester and amide bonds present in the PVP structure.^[^ ^61^ ^]^ As the PVP fragments become smaller and more dispersed in water, they can become more accessible to various microorganisms and enzymes present in the environment and biological system. Similarly, the chitosan biopolymer is a well‐known biocompatible and biodegradable substrate used in various fields.^[^ ^62^ ^]^ The device completely dissolves in water with the biodegradable substrate within 300 s (Figure 8c). The short degradation time gives an opportunity to further control the degradation time of the device by using an encapsulation layer inside the device. In order to assess the performance comparison of the resistive memory in our PTM with several related studies reported previously, a table in the supplementary information (Table S1, Supporting Information) is included. As can be seen, the DC switching response of the device, in terms of ON/OFF ratio, multilevel memory, and longer data retention, are greatly enhanced in our device. The multilevel memory behavior was observed only in our work with distinguishable memory states. Also, neuromorphic characteristics like pulse amplitude variation, pulse width variation, learning behavior, LTP, and LTD demonstrate very satisfactory results, revealing devices could also be employed for neuromorphic computing and deep neural network applications, which have not yet been addressed. Our results open the door to utilizing the W/Mg/PVP/Mg/CHS memristor for neuromorphic computing and integrating its wearable, biocompatible properties into future environmentally‐friendly electronics.

## 3 Conclusion

In summary, we were able to successfully demonstrate the multilevel‐operative physically transient memristor (W/Mg/PVP/Mg/CHS) by tuning the compliance current value. The *I*–*V* curve of the PTM exhibits multilevel RESET‐dependent SET characteristics that vary depending on the compliance current applied (10 µA to 1 mA). The multilevel states result from the metallic filament formed through the electrochemical metallization process of magnesium in the active layer. The device enters the SET state during a positive bias and the RESET state during a negative bias. The PTM exhibits stable switching properties (up to 100 sweeps) with a remarkably high dynamic ON/OFF state resistance ratio (10^6^ Ω) and memory retention time beyond 10^4^ s. The pulse transient measurements demonstrate the device's neuromorphic computation capabilities, resembling the biological function of neurons, making it a potential candidate. Additionally, the device is observed to completely dissolve within 10 min when immersed in DI water, making it a promising candidate for physically transient electronics for security and biosurgery applications. Overall, the findings highlight insightful information about the potential use of our device in healthcare and transient electronics.

## 4 Experimental Section

### Materials

Polyvinylpyrrolidone (PVP) and chitosan (CHS) (pellets) were purchased from Sigma–Aldrich with molecular weights of 10 000 and 50 000–190 000, respectively, and used as is. Magnesium (Mg) and tungsten (W) target metals were purchased from Alfa Aesar 99.99% iTasco, South Korea, and used without any further purification. Acetonitrile (ACN), anhydrous grade, 99.8% from Alfa Aesar, and acetic acid, extra pure from Daejung Pvt. Ltd. used as solvents for electrolyte preparation.

### Device Fabrication

The metal‐insulator‐metal (MIM) array structure device was fabricated on a chitosan‐based biodegradable substrate. The chitosan substrate was prepared by dissolving 0.5 g of chitosan in a 2n acetic acid solution and allowing it to stir for 12 h at 50 °C. The obtained solution was then drop‐cast in a petri dish and allowed to dry at 50 °C. The film was subsequently cut into 1.5 × 1.5 cm^2^ pieces for the fabrication of a physically transient device. The bottom electrode, consisting of Mg with a thickness of ≈180 nm, was deposited on the substrate using the E‐beam evaporation masking technique. The thin layer of PVP was evenly formed using the spin coating method and used as an ion‐conducting solid polymer layer sandwiched between the top and bottom metal electrodes. The polymer electrolyte was prepared by taking a solute‐to‐solvent ratio of 4 wt.% (after optimizing the polymer‐to‐solvent ratio) and stirring vigorously for 2 h at 50 °C followed by ultrasonication. The 0.5 ml of prepared solution was filtered with a 0.2 µm syringe filter and spin‐coated at a speed of 3000 rpm for 60 s to form a ≈230 nm uniform film on the bottom electrode. The film was then vacuum dried continuously for 6 h to obtain a low roughness surface and remove the unwanted solvent's residue. The Mg as a top electrode (≈210 nm) was deposited similar to the bottom electrode using a shadow mask. An ultrathin (≈5 nm) tungsten (W) metal was deposited on the top electrode (Mg) as a protective layer.

### Device and Material Characterization

The amorphous nature of the PVP was analyzed using powder X‐ray diffraction (XRD) Panalytical Empyrean, 2θ ranging from 10° to 80° at 3θ/min. The roughness was measured using an atomic force microscope (AFM NX‐10) by Park Systems. To examine the surface morphology of the polymer matrix and cross‐section of the cell, field emission scanning electron microscopy (FE‐SEM) with a Hitachi SU8010 working at an accelerating voltage of 15 kV was used. The depth profile was performed using a Vacuo X‐ray photoelectron spectroscopy (in vacuo XPS, Ulvac‐PHI Inc. PHI 5000 Versa Probe III). Electrical characterizations were performed using the Keithley (4200A‐SCS, 2635B) semiconductor characterization system. The *I*–*V* characterization of the device was carried out using a MS‐Tech substation with tungsten (W) microprobes, in which bias voltage was applied to the TE swept with a constant sweep rate while the BE was grounded in all cases at air and room temperature.

## Conflict of Interest

The authors declare no conflict of interest.

## Supporting information

Supporting Information

## Data Availability

The data that support the findings of this study are available from the corresponding author upon reasonable request.
