# Supplementary material for: Multilevel Reset Dependent Set of a Biodegradable Memristor with Physically Transient
Source: Adv Sci (Weinh). 2023 Nov 30;11(4):2306206. doi: 10.1002/advs.202306206 (PMC10811477; doi:10.1002/advs.202306206)
Supplement: Supplementary file 1 — Supporting Information [file ADVS-11-2306206-s001.pdf]

ADVANCED SCIENCE
Open Access

Supporting Information

for *Adv. Sci.*, DOI 10.1002/advs.202306206

Multilevel Reset Dependent Set of a Biodegradable Memristor with Physically Transient

*Mohammad Tauquir Alam Shamim Shaikh, Tan Hoang Vu Nguyen, Ho Jung Jeon, Chowdam Venkata Prasad, Kyong Jae Kim, Eun Seo Jo, Sangmo Kim and You Seung Rim**

Supporting Information

Multilevel RESET Dependent SET of a Biodegradable Memristor with Physically Transient

Mohammad Tauquir Alam Shamim Shaikh[1,2], *Tan Hoang Vu Nguyen*[2], *Ho Jung Jeon*[1,2], *Chowdam Venkata Prasad*[2], *Kyong Jae Kim*[2], *Eun Seo Jo*[1], *Sangmo Kim*[2], *You Seung Rim*[1,2]*

[1]*Department of Semiconductor Systems Engineering and Institute of Semiconductor and System IC, Sejong University, Seoul, 05006, Republic of Korea.*

[2]*Department of Intelligent Mechatronics Engineering and Convergence Engineering for Intelligent Drone, Sejong University, Seoul, 05006, Republic of Korea.*

*To whom correspondence should be addressed, *E-mail: youseung@sejong.ac.kr*

1. Conduction Mechanism:

Two different device structures, W/Au/PVP/Au/CHS and W/Mg/PVP/Au/CHS, were fabricated to verify the formation of magnesium based metallic filaments. The device employing gold (Au)-based top and bottom electrodes exhibits no discernible resistive switching behavior. However, when the top electrode was changed to Mg, resistive switching was observed in the device (Figure S4c, Supporting Information). The temperature-dependent study (Figure S4d, Supporting Information**)** revealed metallic behavior, with a decrease in resistance observed in the SET condition as the temperature was decreased in steps (Figure S4d, Supporting Information**).** This decrease in resistance indicated a positive temperature coefficient of resistance for the metal. The similar charge transportation mechanism of filament formation has been described in the Au/Mg/fibroin/Mg and Mg/aloe polysaccharides/ITO works,[1,2] which summarizes the redox-based switching phenomenon.[3–6] As it can be seen, the results in works[1,2] support our experiment data[7] and schematic of the filament formation

& rupture as shown **Figure 4**a and d. In conducting bridge resistive switching memory, the metal filament is surrounded by a dielectric medium, which acts as an electrolyte most of the time, making the redox mechanism of metallic ions the primary consideration.[8]

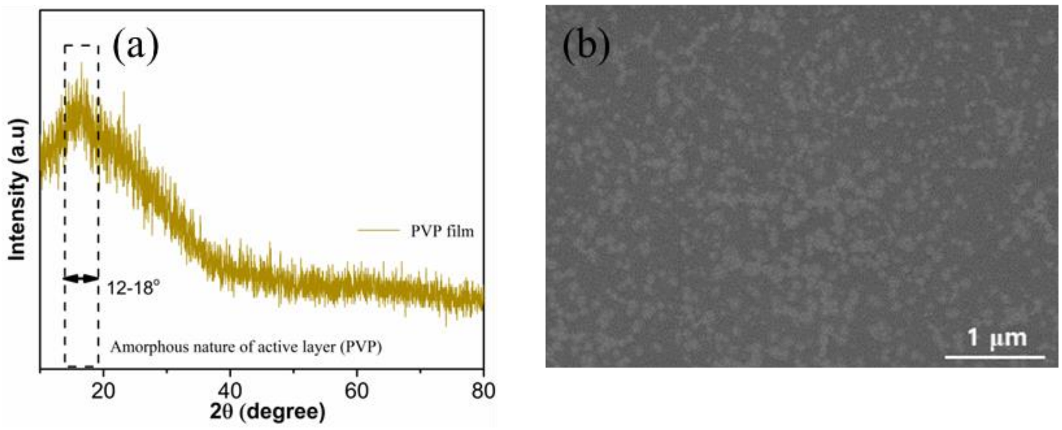

Figure S1. a) X-ray diffraction (XRD) spectra of the PVP active layer. b) Surface morphology of the same active layer.

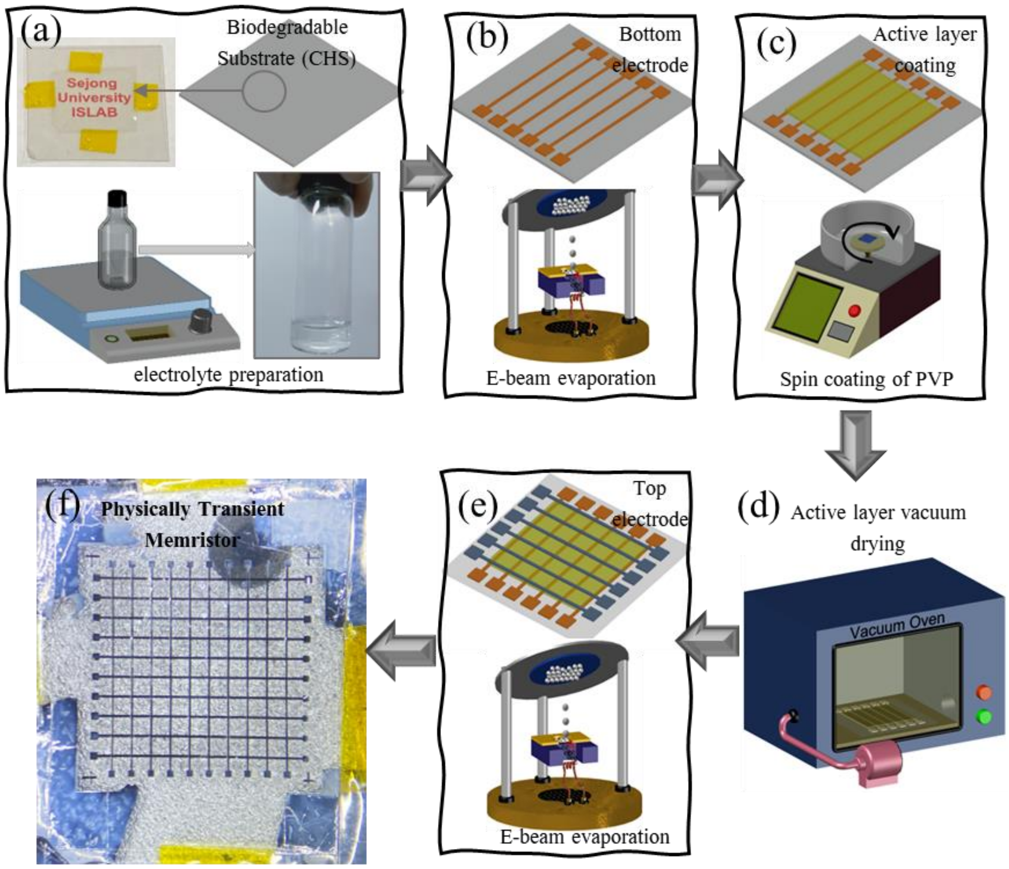

Figure S2. Schematic representation of the fabrication procedure of W/Mg/PVP/Mg/CHS memristor. a) Preparation of PVP solution and substarte. b) Depositing bottom electrode by the e-beam evaporation method. c) Spin coating of PVP on the top of the bottom electrode. d) Vacuum oven drying process after spin coating at 10^{-3} mbar. e) Depositing top electrode and protecting layer by e-beam evaporation and the face target sputter method, respectively. f) 10 x 10 array structure fabricated on chitosan substrate.

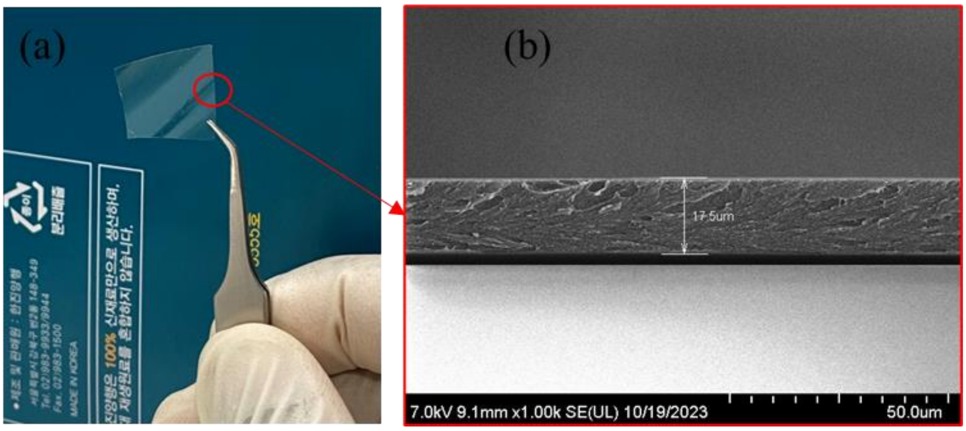

Figure S3. a) Photograph image of biocompatible and biodegradable substrate before fabricating device. b) Substrate FE-SEM cross-section image for thickness measurements (17.5 µm).

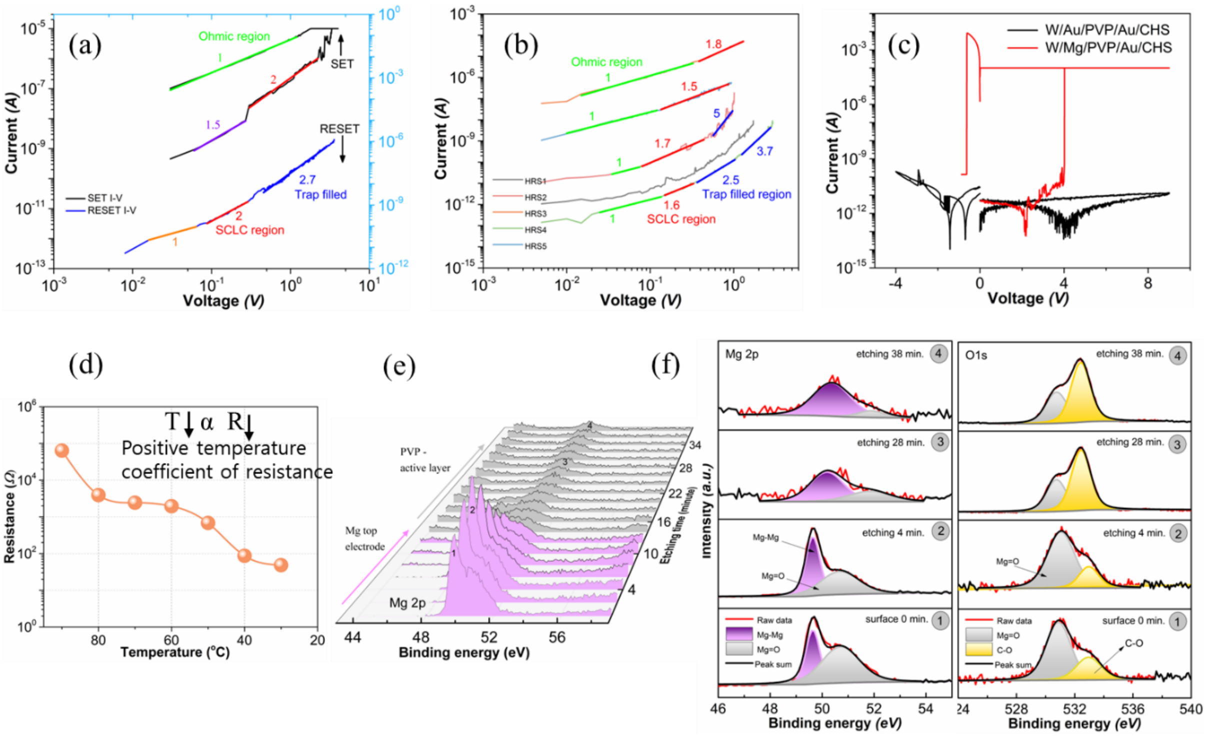

Figure S4. a) Logarithmic plot of I-V, illustrating the slope values of linear fitted curves at positive and negative bias. b) Logarithmic plots of I-V curves at different HRS, illustrating the slope values of linear fitted curves at positive bias. c) I - V response of the W/Au/PVP/Au/CHS and W/Mg/PVP/Au/CHS devices. d) Impact of temperature in the range of 30 to 90 °C after the LRS. e,f) depth profile analysis (Mg spectra) and deconvoluted Mg 2p and O1s XPS spectra, respectively.

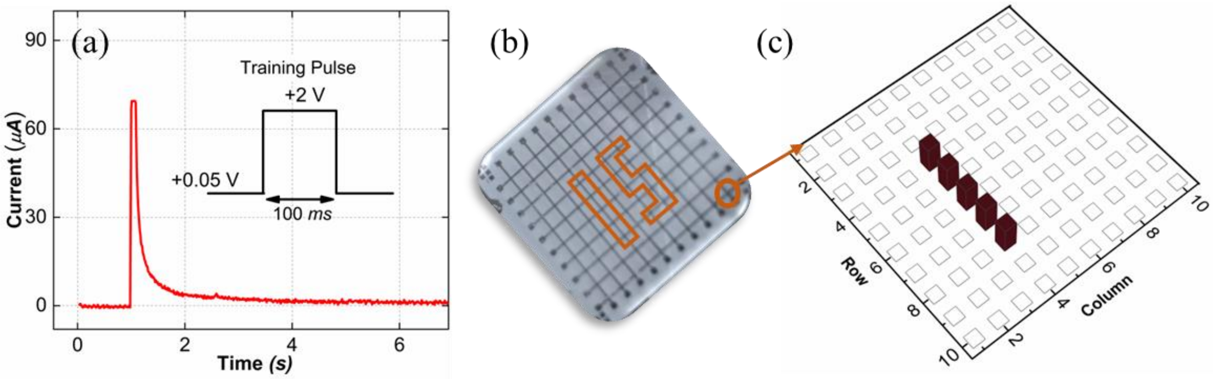

Figure S5. learning behavior by a) inducing a training pulse (V_{pulse} = 2 V, t_{width} = 100 ms). b) 10 x 10 array photograph of PTM. c) Schematic of letter "I" consists of 5 pixels generated by a training pulse to show the learning process in the brain.

Table S1: Performance comparison among the transient and multilevel devices.

Device structure	Resistive switching	Multilevel switching	$R_{ON/OFF}$ (ratio)	Retention (S)	$V_{SET/RESET}$ (V)	References
Ag/PVP/HfO_x/ITO	Bipolar	No	-	7.2 x 10^3	1.03/-0.68	[9]
Al/GO:PVP/PEDOT:PSS/ITO	Bipolar	No	10^2	1 x 10^4	2.3 to 3.5/--0.7 to -0.2	[10]
Al/WS_2 QD-PVP/ITO	Bipolar	No	-	1.2 x 10^4	0.7/-3	[11]
ITO/ Ti_3C_2@PVPy/Al	Bipolar	Yes	10^4	5 x 10^4	0.54/-2.49	[12]
Mg/fibroin/Mg	Bipolar	No	10^2	10^4	0.7 to 1.7 / -0.5 to -1	[1]

Mg/Ag- doped chitosan/Mg	Bipolar	No	-	10^4	1.63/-0.82	[13]
Al/PVP–NCQD/ITO	Bipolar	No	10^4	-	~1.8/-1	[14]
Al/MoS_2-PVP/rGO	Bipolar	No	10^2	-	3.5/-4.5	[15]
**W/Mg/PVP/Mg/CHS**	**Bipolar**	**Yes**	**10^6**	**10^4**	**2.41/-1.62**	**This work**

References:

[1] H. Wang, B. Zhu, X. Ma, Y. Hao, X. Chen, *Small* **2016**, *12*, 2715.

[2] Z. X. Lim, K. Y. Cheong, *Adv. Mater. Technol.* **2018**, *3*, 1.

[3] B. Cho, J.-M. Yun, S. Song, Y. Ji, D.-Y. Kim, T. Lee, *Adv. Funct. Mater.* **2011**, *21*, 3976.

[4] X. Guo, C. Schindler, S. Menzel, R. Waser, *Appl. Phys. Lett.* **2007**, *91*, DOI 10.1063/1.2793686.

[5] J. Zhao, H. Sun, S. Dai, Y. Wang, J. Zhu, *Nano Lett.* **2011**, *11*, 4647.

[6] Y. Hirose, H. Hirose, *J. Appl. Phys.* **1976**, *47*, 2767.

[7] A. Gumyusenge, A. Melianas, S. T. Keene, A. Salleo, *Annu. Rev. Mater. Res.* **2021**, *51*, 47.

[8] Z. Wang, F. Zeng, J. Yang, C. Chen, F. Pan, *ACS Appl. Mater. Interfaces* **2012**, *4*, 447.

[9] I. Varun, D. Bharti, A. K. Mahato, V. Raghuwanshi, S. P. Tiwari, *Solid State Ionics* **2018**, *325*, 196.

[10] W. K. Kim, C. Wu, T. W. Kim, *Appl. Surf. Sci.* **2018**, *444*, 65.

[11] H. An, Y. H. Lee, J. H. Lee, C. Wu, B. M. Koo, T. W. Kim, *Sci. Rep.* **2020**, *10*, 1.

[12] G. Ding, K. Zeng, K. Zhou, Z. Li, Y. Zhou, Y. Zhai, L. Zhou, X. Chen, S.-T. Han, *Nanoscale* **2019**, *11*, 7102.

[13] N. R. Hosseini, J.-S. Lee, *Adv. Funct. Mater.* **2015**, *25*, 5586.

[14] T. Zeng, Z. Yang, J. Liang, Y. Lin, Y. Cheng, X. Hu, X. Zhao, Z. Wang, H. Xu, Y. Liu, *Nanoscale Adv.* **2021**, *3*, 2623.

[15] J. Liu, Z. Zeng, X. Cao, G. Lu, L. H. Wang, Q. L. Fan, W. Huang, H. Zhang, *Small* **2012**, *8*, 3517.
